# Intraprocedural echocardiographic guidance in transcatheter mitral valve interventions: from procedure-specific workflows to integrated multimodal platforms

**DOI:** 10.1186/s44348-026-00092-7

**Published:** 2026-09-08

**Authors:** Stan A. Benjamins, Martijn G. H. Vrijkorte, Leo Timmers, Benno J. W. M. Rensing, Martin J. Swaans

**Affiliations:** Department of Cardiology, St. Antonius Hospital, Nieuwegein, The Netherlands

**Keywords:** Transcatheter mitral valve interventions, Intraprocedural echocardiography, Transesophageal echocardiography, Multiplanar reconstruction, Echocardiography-fluoroscopy fusion, Intracardiac echocardiography, Artificial intelligence, Multimodal imaging guidance

## Abstract

Mitral regurgitation (MR) is the most common left-sided valvular heart disease worldwide and carries significant morbidity and mortality when untreated. The expanding use of transcatheter mitral valve interventions (TMVIs), including mitral transcatheter edge-to-edge repair (M-TEER) and transcatheter mitral valve replacement (TMVR), to anatomically complex, high-risk patient populations has substantially increased the demands on intraprocedural echocardiographic guidance. Yet current consensus documents provide modality-level guidance without explicitly formalizing the divergent imaging requirements inherent to these two mechanistically distinct interventions. This state-of-the-art review synthesizes evidence on intraprocedural echocardiographic guidance for TMVIs, integrating current data on 2D and 3D transesophageal echocardiography, real-time multiplanar reconstruction (MPR), echocardiography-fluoroscopy fusion imaging, 3D intracardiac echocardiography, and artificial intelligence (AI)-assisted decision-support tools. Two fundamentally distinct imaging paradigms are identified and formalized: a leaflet-level, temporally driven framework for M-TEER and an annular-level, spatially driven framework for TMVR. These paradigms reflect divergent anatomical targets and imaging priorities, with concrete implications for workflow design, operator-echocardiographer communication, and complication avoidance. Real-time MPR serves as the geometric backbone bridging 2D temporal precision with 3D spatial clarity. AI-assisted tools provide a computational guidance layer enhancing spatial consistency and procedural reproducibility while preserving, and not replacing, expert echocardiographic judgment. Effective intraprocedural echocardiographic guidance is objective-driven rather than modality-centric. The modalities described in this review function not as interchangeable alternatives but as complementary layers of a coherent, workflow-integrated architecture. The transition toward integrated, platform-based multimodal guidance defines the trajectory of next-generation TMVI echocardiography, requiring rigorous prospective validation, protocol standardization across device platforms, and training frameworks that reflect the multimodal nature of modern structural practice.

## Background

Mitral regurgitation (MR) is the most prevalent left-sided valvular heart disease worldwide, with clinically significant moderate-to-severe MR affecting an estimated 1.5% of the adult population, a burden that rises sharply with advancing age and carries significant morbidity and mortality when left untreated [1]. Despite this burden, a substantial proportion of symptomatic patients with severe MR remain undertreated due to high or prohibitive surgical risk, underscoring the need for effective transcatheter alternatives [2–4].

Over the past decade, transcatheter mitral valve interventions (TMVIs) have evolved from high-risk feasibility procedures to increasingly standardized therapies with a robust evidence base [5–8]. Mitral transcatheter edge-to-edge repair (M-TEER) has demonstrated durable MR reduction and sustained reductions in all-cause mortality and heart failure hospitalization at 5 years in the COAPT trial [5, 6]. These findings have been further validated by the RESHAPE-HF2 and MATTERHORN trials, which demonstrated superiority over guideline-directed medical therapy in moderate-to-severe functional MR and noninferiority to surgical repair in secondary MR, respectively [7, 8]. In parallel, transcatheter mitral valve replacement (TMVR) has transitioned from investigational feasibility to approved clinical practice [9]. The transapical Tendyne system (Abbott) received US Food and Drug Administration approval for patients with symptomatic MR and significant mitral annular calcification (MAC) ineligible for surgery or TEER [10], while the transseptal SAPIEN M3 system (Edwards Lifesciences) became the first fully percutaneous TMVR device to achieve regulatory clearance [11]. The 2025 European Society of Cardiology (ESC) and the European Association for Cardio-Thoracic Surgery (EACTS) Guidelines formalize this progress, assigning M-TEER to a Class I, Level A recommendation in carefully selected patients with ventricular secondary MR who remain symptomatic despite guideline-directed medical therapy, while distinguishing this population from atrial secondary MR, in whom treatment selection remains more individualized [4].

As the therapeutic landscape expands to anatomically complex and heterogeneous patient cohorts, procedural demands have intensified correspondingly. The mitral valve apparatus presents inherent 3D complexity: a nonplanar saddle-shaped annulus, dynamic leaflet excursion throughout the cardiac cycle, intimate spatial relationships with the left ventricular outflow tract (LVOT) and aortic root, and highly variable patterns of annular remodeling, calcification, and chordal architecture [12, 13]. In secondary MR, left ventricular remodeling, papillary muscle displacement, and progressive annular dilatation compound these structural challenges, making real-time echocardiographic assessment of device-tissue interaction not merely supportive but operationally indispensable for safe and effective procedural execution [3, 14].

Fluoroscopy remains essential for catheter navigation, device deployment, and calcification landmark identification, but cannot meet the soft-tissue imaging demands of modern TMVIs. Its inability to visualize leaflet scallops, the coaptation line, commissural morphology, chordal structures, or the annular-LVOT relationship renders it insufficient as the sole guidance modality as procedural complexity increases [15, 16]. Echocardiography has therefore emerged as the primary intraprocedural guidance modality, providing continuous anatomical visualization, hemodynamic assessment, and evaluation of device-tissue interactions [9, 13, 15]. Advances in 3D transesophageal echocardiography (TEE), multiplanar reconstruction (MPR), echocardiography-fluoroscopy fusion imaging, 3D intracardiac echocardiography (ICE), and artificial intelligence (AI)-assisted decision-support tools have substantially expanded echocardiographic capabilities. Critically, their effectiveness increasingly depends not on individual modalities alone, but on their structured integration into coherent, workflow-driven strategies that support continuous spatial orientation and real-time decision-making throughout each procedural phase.

Despite this rapid evolution, current consensus documents and imaging guidelines provide modality-level guidance without explicitly formalizing the divergent procedural imaging requirements of leaflet-level repair versus annular-level replacement. The emerging roles of AI-assisted guidance, platform-based multimodal integration, and device-specific imaging protocols within structured intraprocedural workflows remain similarly unaddressed [12, 13]. This state-of-the-art review addresses that gap by formalizing a clinically consequential yet underrecognized distinction: M-TEER and TMVR require fundamentally different imaging paradigms [9, 17]. Within this conceptual framework, this review synthesizes the evidence base for contemporary echocardiographic modalities, proposes a structured stepwise imaging framework applicable across TMVI platforms, and examines how AI-assisted tools and integrated imaging environments are shaping next-generation procedural reproducibility and scalability.

## Intraprocedural echocardiographic guidance modalities

Echocardiography has evolved from a primarily diagnostic modality into the central framework for intraprocedural guidance in TMVIs, providing real-time anatomical navigation, dynamic assessment of device-tissue interactions, and immediate hemodynamic evaluation throughout each procedural phase [9, 15]. Contemporary intraprocedural echocardiography functions as a multimodal approach in which complementary techniques are selectively combined based on procedural demands and anatomical context. The concepts and imaging objectives described in this review are applicable across platforms, although access to advanced integrated imaging environments, including AI-assisted guidance, may be subject to institutional resource constraints and vendor availability. An overview of the strengths, limitations, and workflow roles of each modality is presented in Table 1.

**Table 1:** Strengths, limitations, and intraprocedural workflow role of echocardiographic guidance modalities in TMVIs

Modality	Principal strength	Key limitation	Primary intraprocedural role
2D TEE	Highest temporal resolution; continuous real-time visualization; standardized acquisition planes; Doppler assessment of residual MR and gradients	Planar imaging only; cognitive 3D reconstruction required; susceptible to out-of-plane motion; limited spatial context in complex anatomy	Real-time catheter and device tracking; functional Doppler assessment; temporal reference standard throughout all procedural phases
3D TEE	*En face* mitral valve visualization; volumetric spatial orientation; scallop and commissural identification; annular geometry appreciation	Lower temporal resolution; acoustic window dependency; stitching/motion artifacts in atrial fibrillation	Global anatomical orientation; device-leaflet spatial alignment; annular geometry and device seating; coaptation geometry assessment
Real-time MPR	Geometrically precise orthogonal planes; probe-independent alignment; bridges 2D temporal precision with 3D spatial clarity	Dependent on 3D acquisition quality; cardiac rhythm stability required; substantial operator expertise needed	Geometric backbone of TMVI guidance; reproducible annular-plane alignment; continuous orthogonal assessment of device trajectory
Echocardiography-fluoroscopy fusion	Unified crossmodal spatial reference; reduces modality-switching burden; potential reduction in fluoroscopy time	Susceptible to co-registration drift; requires recalibration; does not generate new anatomical data; adds workflow complexity	Crossmodality spatial integration; anatomical landmark projection; support for coordinated operator-echocardiographer communication
3D ICE	No esophageal intubation or general anesthesia required; near-field visualization of septal tenting; expands procedural accessibility	Limited field of view; highly operator-dependent; additional vascular access required; predominantly feasibility-level evidence	Selective adjunct in TEE-contraindicated patients; transseptal access guidance; near-field device-tissue visualization
AI-assisted tools	Automated landmark tracking; standardized geometric reference frames; reduced cognitive load; supports procedural standardization	Image quality-dependent; limited prospective comparative data; vendor-specific implementations; emerging evidence base	Computational guidance layer; persistent spatial orientation; support for procedural reproducibility and operator training

AI, artificial intelligence; ICE, intracardiac echocardiography; MPR, multiplanar reconstruction; MR, mitral regurgitation; TEE, transesophageal echocardiography; TMVI, transcatheter mitral valve intervention

### 2D and 3D TEE

The 2D TEE remains the foundational imaging modality for intraprocedural guidance in TMVIs, with high temporal resolution enabling continuous real-time visualization of catheter motion, leaflet dynamics, and device-tissue interaction during time-sensitive and motion-dependent intraprocedural steps [15, 18]. Well-established standardized acquisition planes (mid-esophageal four-chamber, long-axis, commissural, and bicaval views) support reproducible intraprocedural communication between echocardiographer and interventional operator [18].

The intrinsic limitation of 2D TEE is its planar representation of a 3D structure. Accurate spatial interpretation requires cognitive reconstruction of anatomical relationships across sequential imaging planes, which is a process prone to error in complex settings including annular remodeling, asymmetric leaflet tethering, or calcification [19, 20]. Spatial accuracy may be further compromised by probe angulation, out-of-plane motion, and foreshortening during critical decision-making [21].

The 3D TEE addresses these limitations by providing volumetric datasets enabling anatomically intuitive visualization [20, 22]. *En face* views from both the left atrial and ventricular perspectives facilitate scallop identification, commissural alignment, and coaptation geometry assessment [20, 23]. Volumetric rendering improves appreciation of annular geometry, device seating, and the spatial relationship between prosthesis and LVOT [9, 22]. Contemporary matrix-array probes provide full-volume, zoom, and biplane acquisition modes, with vendor-specific temporal and spatial resolution profiles that influence acquisition strategy selection across procedural phases [19, 24].

The principal trade-off of 3D TEE is its lower temporal resolution, which reduces imaging fidelity during rapid leaflet motion or dynamic device maneuvers. Motion and stitching artifacts remain a clinically relevant limitation in atrial fibrillation, a frequent TMVI comorbidity (> 50%), where sequential-beat volume compositing is compromised. Single-beat acquisition modes at reduced sector width partially mitigate this limitation [19, 21, 25]. Accordingly, 2D and 3D TEE function as complementary rather than interchangeable modalities: 2D TEE provides temporal precision and functional assessment, whereas 3D TEE provides spatial context and anatomical characterization [15, 20]. The transition toward 3D-centered guidance, with real-time MPR as the operational interface, represents the most important shift in contemporary structural echocardiography practice.

### Real-time MPR

Real-time MPR converts 3D volumetric echocardiographic datasets into multiple simultaneously displayed orthogonal imaging planes, each independently adjustable without requiring probe repositioning [26, 27]. Standard configurations provide two orthogonal long-axis planes, a short-axis plane, and a co-registered 3D reference rendering, enabling comprehensive visualization of the mitral valve apparatus, annular geometry, and device trajectory from a single acquisition [26, 28]. Implementation varies across vendor platforms (Philips Healthcare, Siemens Healthineers, GE Healthcare), and operators should be familiar with the specific display conventions and adjustment tools of their local system.

Unlike sequentially acquired 2D planes or static 3D renderings, MPR-derived planes maintain true orthogonality relative to anatomical landmarks throughout the cardiac cycle, providing stable, reproducible spatial reference frames independent of probe orientation [26, 27]. This substantially reduces reliance on cognitive spatial reconstruction and mitigates the key limitations of both 2D and 3D TEE when used in isolation. In transseptal guidance specifically, MPR allows simultaneous orthogonal visualization of puncture height, anteroposterior position, and septal thickness from a single acquisition, a task requiring multiple sequential probe rotations with standard 2D imaging alone [29, 30].

MPR functions not as a standalone modality but as a workflow-integrated processing layer that converts existing volumetric datasets into geometrically stable, orthogonally aligned views, bridging the temporal precision of 2D TEE with the spatial clarity of 3D TEE [26, 27, 31]. Nevertheless, its effectiveness is contingent on the quality of the underlying acquisition, which remains dependent on acoustic windows, acquisition settings, and cardiac rhythm stability [19, 26]. Effective use requires dedicated expertise in volumetric acquisition optimization, plane selection, and orthogonality maintenance. These competencies should be considered core curriculum for structural echocardiographers rather than advanced optional skills [26, 27].

MPR thus represents a shift from modality-based imaging toward structured, workflow-integrated spatial guidance. By providing reproducible orthogonal orientation and a shared geometric framework, it constitutes the geometric backbone of advanced intraprocedural guidance [26, 27]. This spatial framework is the foundation upon which echocardiography-fluoroscopy fusion imaging and AI-assisted spatial referencing are built.

### Echocardiography-fluoroscopy fusion imaging

Echocardiography-fluoroscopy fusion imaging co-registers real-time echocardiographic data, encompassing 2D planes, 3D renderings, and MPR-derived spatial frameworks, with the fluoroscopic projection within a shared spatial coordinate system [15, 32]. Soft-tissue anatomical landmarks are projected directly onto the fluoroscopic image, combining high-resolution structural visualization with stable catheter and device representation.

The principal benefit of fusion imaging is reduction of the cognitive burden associated with translating echocardiographic spatial information into the fluoroscopic field of view [15, 16]. During conventional guidance, echocardiographers and interventional operators each interpret their respective imaging streams separately, requiring continuous verbal communication and cognitive integration of complementary but spatially independent displays. Fusion imaging replaces this fragmented workflow with a common visual reference, supporting more intuitive assessment of catheter trajectory, device alignment, and target engagement [32, 33]. Reported procedural benefits include reductions in fluoroscopy time and radiation exposure during M-TEER guidance, as demonstrated in a structured protocol by Melillo et al. [33]. However, these findings derive from observational single-center series and should be regarded as supportive rather than definitive. No randomized controlled trial has evaluated fusion imaging as an independent procedural variable, and effect sizes vary substantially across centers and device platforms [15, 32].

The reliability of fusion imaging is critically dependent on the accuracy of co-registration and its maintenance throughout the intervention. Co-registration drift, arising from transducer repositioning, patient movement, respiratory excursion, or system calibration loss, may progressively degrade spatial alignment and necessitate recalibration [32, 33]. Importantly, fusion imaging does not generate new anatomical information. In contemporary practice, it is best understood as an adjunctive spatial integration tool rather than a primary guidance modality. By projecting echo-defined landmarks directly into the fluoroscopic field, it reduces the cognitive cost of modality switching at procedurally critical moments, specifically catheter advancement, device orientation, and final positional confirmation, representing a functional bridge toward the fully integrated platform-based guidance [15, 32, 33]. This role applies across both M-TEER and TMVR, though the published procedural evidence is predominantly derived from M-TEER experience [33].

#### 3D ICE

The 3D ICE enables real-time volumetric intracardiac visualization through catheter-mounted matrix-array transducers, without esophageal intubation or general anesthesia [34, 35]. Advances in catheter-based ultrasound technology, exemplified by commercially available platforms including VeriSight Pro (Philips Healthcare), AcuNav Volume (Siemens Healthineers), and NuVera (Biosense Webster), have expanded ICE from conventional single-plane imaging to full volumetric acquisitions supporting MPR and 3D rendering from intracardiac vantage points [35, 36].

The principal advantage of 3D ICE is procedural accessibility, particularly in patients in whom TEE is not feasible or desirable. This includes individuals with esophageal pathology, such as varices, strictures, or prior esophageal surgery, as well as patients with TEE intolerance, or preference for conscious sedation. A 3D ICE may expand imaging options and enable procedures that would otherwise require general anesthesia [34, 35, 37]. This is an important consideration in the elderly, frail, and high-risk populations who constitute the majority of TMVI candidates. Near-field visualization of the interatrial septum offers a distinct advantage in patients where esophageal probe positioning is suboptimal or TEE is contraindicated, though it does not replicate the simultaneous orthogonal assessment achievable with MPR-based TEE [36]. The position statements from the Society for Cardiovascular Angiography & Interventions (SCAI) and the *Journal of the American College of Cardiology* (JACC) Cardiovascular Imaging have both endorsed selective 3D ICE use in structural interventions, with appropriate operator competency standards [34, 37].

The limitations of 3D ICE in the TMVI context must be clearly understood. The field of view is constrained by catheter proximity and imaging depth (typically 8–10 cm), limiting comprehensive visualization of the global mitral valve apparatus, annular geometry, and spatial relationships between the prosthesis and LVOT compared with TEE [36, 38]. Image quality is highly dependent on catheter stability and orientation, and volumetric acquisition is susceptible to motion artifacts during catheter manipulation. Additional femoral venous access and intracardiac catheter navigation are required, increasing procedural complexity, vascular risk, and disposable cost [35, 36]. Importantly, current evidence for 3D ICE in TMVI consists primarily of case reports, feasibility studies, and single-center observational series [35, 36].

Compared with TEE, 3D ICE provides less comprehensive anatomical context for the full mitral apparatus, particularly regarding *en face* visualization, annular geometry assessment, and the stable spatial reference frames required across multiple procedural phases [36, 38]. Although MPR can be applied to ICE-derived volumes, spatial coverage and geometric consistency remain inferior to transesophageal acquisitions for this purpose. The cognitive and technical demands of catheter-based volumetric guidance are also distinct from TEE. Precise catheter positioning during concurrent procedural activity, combined with real-time 3D interpretation from non-standard imaging planes, constitutes a dedicated learning curve that should not be conflated with TEE competency [35].

The 3D ICE is therefore best conceptualized as a complementary, patient-selective imaging option that expands procedural accessibility in specific clinical scenarios rather than as a replacement for established TEE-, MPR-, and fusion-based workflows [34, 35, 37]. Future prospective comparative data will be essential to define the scope and conditions under which 3D ICE guidance can independently support complex TMVI.

### AI-assisted decision-support tools

AI-assisted decision-support tools represent an emerging computational layer within intraprocedural echocardiographic guidance, designed to enhance spatial consistency and procedural reproducibility rather than to replace expert imaging judgment [39, 40]. Unlike the imaging modalities described above, AI tools do not generate new anatomical information. Instead, it operates on existing imaging data, primarily 3D TEE volumes, MPR spatial frameworks, and fusion-registered datasets, to automate landmark tracking, stabilize geometric reference frames, and provide continuous real-time feedback on device position relative to predefined anatomical targets [39–42]. The detailed clinical evidence, procedure-specific roles, platform implementations, and limitations of AI-assisted guidance are addressed comprehensively in AI-Assisted Intraprocedural Guidance: Roles, Evidence, and Limitations section.

## Structured imaging-guided approach to TMVI

TMVIs follow a shared sequence of imaging-guided procedural phases regardless of device type or mechanism of action. At each phase, echocardiography functions not as a passive visualization tool but as an active guidance framework, continuously adapting to evolving anatomical conditions, device-tissue dynamics, and hemodynamic feedback [9, 13]. A structured stepwise approach to this guidance enhances procedural precision, reproducibility, and interdisciplinary coordination, qualities that grow increasingly critical as device platforms diversify, patient anatomies become more complex, and the consequences of spatial misorientation increase correspondingly.

### Transseptal puncture

Transseptal puncture (TSP) is the foundational imaging-guided step of all transvenous TMVIs, as its geometry directly determines downstream catheter maneuverability, trajectory control, and device alignment [13, 30]. The core clinical decision is where precisely to puncture the fossa ovalis, balancing adequate left atrial workspace against a stable coaxial delivery trajectory toward the mitral annulus. The optimal puncture height, defined as the distance between the puncture site and the mitral valve coaptation plane, is 4 to 5 cm for M-TEER, providing sufficient room for the steerable guide catheter to maneuver without excessive angulation [30]. For TMVR, which involves larger and stiffer delivery systems, a higher and more posterior puncture site is typically required to maintain coaxiality with the annular plane [9, 13].

The 2D TEE provides continuous real-time visualization through standardized views: the bicaval view for superoinferior axis assessment, the short-axis aortic valve view for anteroposterior positioning, and the four-chamber view for height measurement [18, 30]. TEE-guided TSP substantially improves puncture site accuracy compared with fluoroscopy alone, with prospective data demonstrating elimination of dangerous needle positions under echocardiographic guidance [29, 30]. MPR provides incremental benefit over sequential 2D imaging by enabling simultaneous orthogonal visualization of puncture height, anteroposterior position, and septal thickness from a single acquisition, improving spatial accuracy in anatomically challenging or remodeled septa [29]. Echocardiography-fluoroscopy fusion imaging projects echocardiography-defined septal landmarks onto the fluoroscopic field, enhancing eye-hand coordination during needle advancement [32, 33]. In TEE-contraindicated patients, 3D ICE provides an alternative near-field visualization of septal tenting and needle orientation, though without the simultaneous orthogonal assessment achievable with MPR-based TEE [34, 37].

### Left intra-atrial navigation

Following transseptal access, the core imaging task is establishing and maintaining a geometrically favorable delivery trajectory toward the mitral annular plane [13, 14]. This is straightforward in normal anatomy but demands active echocardiographic management in challenging left atrial geometries. Left atrial enlargement distorts the geometric relationship between the TSP site and the mitral annular plane, increasing the risk of noncoaxial approach vectors. Prior catheter ablation or iatrogenic atrial septal defect closure may create unpredictable catheter deflection patterns. In these scenarios, MPR-derived orthogonal views aligned with the intended delivery axis maintain true orthogonality to the target regardless of probe position or left atrial distortion, feedback that sequential 2D plane acquisition cannot replicate [26, 27]. This reduces reliance on cognitive spatial reconstruction and enables reproducible trajectory adjustments in both mediolateral and anteroposterior axes.

The 2D TEE provides continuous high-temporal-resolution monitoring of catheter advancement, enabling immediate correction of suboptimal trajectories [15]. The 3D TEE adds volumetric context for appreciating the optimal approach vector in enlarged or distorted atria [9, 14]. Echocardiography-fluoroscopy fusion imaging maintains a shared anatomical reference throughout navigation, supporting coordinated decision-making between the echocardiographer and interventional operator [32, 33]. AI-assisted platforms can continuously track catheter position relative to predefined target zones and provide real-time visual feedback on trajectory deviation relative to annular geometry [41, 42].

### Device positioning and orientation at the mitral annulus

As the device approaches the mitral annulus, imaging guidance shifts from trajectory control to precise geometric alignment with leaflet scallops, the commissural axis, and the coaptation line [13, 15, 30]. Even minor deviations in depth, rotation, or mediolateral position may compromise engagement, predispose to noncoaxial deployment, or risk interaction with the LVOT, necessitating continuous spatial reassessment within the dynamic annular environment.

The 2D TEE enables real-time assessment of device depth and mediolateral position. The 3D TEE provides *en face* visualization of the device relative to scallops and commissures, facilitating rotational alignment assessment and evaluation of annular conformity [20, 28]. Real-time MPR enhances predeployment control by displaying orthogonal views aligned with both the device axis and the annular plane, allowing confirmation of perpendicularity and symmetry without reliance on cognitive spatial reconstruction [26, 28, 31]. Echocardiography-fluoroscopy fusion maintains spatial reference during fine positional adjustments, minimizing modality switching and preserving procedural context. AI-assisted tools may provide continuous feedback on translational and rotational alignment relative to annular landmarks [41, 42].

### Leaflet or annular engagement during device deployment

Device deployment is the most time-sensitive and technically demanding phase, requiring simultaneous real-time structural visualization and hemodynamic feedback to guide tissue engagement and detect inadequate capture before the device is released [13, 15, 30]. Variability in leaflet morphology, mobility, calcification, and annular geometry may necessitate real-time adjustments during deployment. Successful engagement ensures secure seating, restoration of leaflet coaptation or annular support, and minimization of residual regurgitation.

The 2D TEE provides high frame-rate visualization of leaflet capture, insertion depth, and symmetry, enabling immediate detection of incomplete or asymmetric engagement. The 3D TEE offers a global *en face* perspective of device-valve interaction, facilitating identification of residual coaptation gaps, malposition, or asymmetric seating that may not be apparent on planar imaging [20, 22]. Real-time MPR simultaneously displays orthogonal views of leaflet capture or annular seating, supporting controlled adjustments with preserved spatial consistency [26, 31]. Echocardiography-fluoroscopy fusion imaging maintains anatomical orientation during deployment when fluoroscopy assumes visual primacy, while 3D ICE provides near-field visualization of tissue-device contact in selected cases [32, 36]. AI-assisted tools can further support this phase by monitoring device stability and symmetry relative to predefined target planes [41, 42].

### Postdeployment structural and functional evaluation

Postdeployment echocardiographic assessment fulfills two critical functions: confirming procedural success and detecting acute complications requiring prompt intervention [9, 15, 30]. Acute complications requiring echocardiographic recognition include LVOT obstruction, device embolization or instability, pericardial effusion and cardiac tamponade, severe residual or worsened MR necessitating repositioning or additional device implantation, and procedure-induced mitral stenosis from excessive transmitral gradient elevation [46, 47]. Recognition of these complications requires active, structured assessment, and echocardiographers should follow a systematic evaluation protocol at this phase regardless of the apparent clinical status.

The 2D TEE with color-flow and spectral Doppler rapidly assesses residual MR severity, paravalvular leak, leaflet mobility, and transmitral and LVOT gradients, all of which constitute the functional endpoints of the intervention [18, 30]. The 3D TEE provides precise *en face* visualization of device seating, annular conformity, and coaptation geometry, supporting accurate localization and quantification of residual defects that may be underestimated on planar imaging [20, 22]. Real-time MPR refines assessment by displaying orthogonal views of transmitral flow jets and coaptation surfaces, enhancing diagnostic confidence [26, 31]. Specifically for TMVR, 3D TEE combined with continuous-wave Doppler plays a unique intraprocedural role in detecting dynamic LVOT obstruction. This may emerge or intensify acutely during device deployment as a result of acute changes in loading conditions, catecholamine use, device depth, anterior leaflet displacement, or systolic anterior motion-like physiology, and may therefore not be fully predicted by static preprocedural computed tomography (CT)-derived neo-LVOT assessment. A prospective study from Bartkowiak et al. [43] demonstrated strong agreement between 3D TEE-derived neo-LVOT predictions and CT-based measurements in patients undergoing TMVR, supporting a complementary intraprocedural role for echocardiography alongside preprocedural CT planning [43].

## Intraprocedural imaging paradigms: M-TEER vs. TMVR

Although M-TEER and TMVR share the foundational procedural phases, their intraprocedural imaging requirements diverge fundamentally [9, 17]. These differences arise from distinct anatomical targets, device mechanics, and procedural objectives, resulting in two contrasting paradigms: a leaflet-level, temporally driven framework for M-TEER and an annular-level, spatially driven framework for TMVR. This distinction, formalized in Table 2, is essential for tailoring imaging workflows, optimizing procedural precision, and preventing complications that arise from applying a uniform approach to mechanistically distinct interventions.

**Table 2:** Comparative imaging paradigms in M-TEER and TMVR

Domain	M-TEER: leaflet-level, temporally driven	TMVR: annular-level, spatially driven
Anatomical target	Mitral leaflet free edges and coaptation line	Mitral annulus, subannular apparatus, and neo-LVOT
Procedural objective	Durable, symmetric leaflet coaptation with MR reduction	Stable, coaxial prosthesis implantation with full expansion and preserved LVOT patency
Dominant imaging priority	High temporal resolution; dynamic assessment of leaflet motion and grasp geometry	High spatial resolution; geometric orientation, annular alignment, and depth control
Critical imaging challenge	Rapid leaflet excursion; heterogeneous morphology; limited left atrial workspace	Irregular or calcified annulus; large stiff delivery systems; LVOT obstruction risk
Key procedural checkpoint	TSP height 4–5 cm; symmetric leaflet insertion; residual MR grade; transmitral gradient	Coaxial alignment; implantation depth; prosthesis expansion; LVOT gradient post deployment
Major imaging-related risk	Single-leaflet device attachment; residual MR; procedure-induced mitral stenosis	LVOT obstruction; prosthesis malposition or embolization; paravalvular leak
Procedural endpoint	Stable clip(s) with effective MR reduction and acceptable transmitral gradient	Stable prosthesis with full expansion, no significant paravalvular leak, and preserved LVOT flow

LVOT, left ventricular outflow tract; MR, mitral regurgitation; M-TEER, mitral transcatheter edge-to-edge repair; TMVR, transcatheter mitral valve replacement; TSP, transseptal puncture

### Mitral transcatheter edge-to-edge repair

#### Leaflet-level, temporally driven guidance

M-TEER requires high-precision imaging of the mitral leaflets within a confined and dynamically changing left atrial workspace [3, 15]. Procedural success depends on achieving stable, symmetric leaflet insertion and sustained coaptation throughout the cardiac cycle. Because mitral leaflet excursion traverses its full range within approximately 120 ms of the cardiac cycle, intraprocedural imaging must prioritize high temporal resolution, real-time grasp geometry assessment, and accurate orientation relative to the coaptation line [18].

These demands create device-specific imaging challenges. Limited left atrial workspace constrains catheter maneuverability, amplifying the importance of precise TSP geometry. Leaflet morphology varies fundamentally between primary and secondary MR: redundant prolapsing tissue in degenerative disease contrasts with tethered, restricted leaflets in functional disease, requiring accurate 3D characterization across the cardiac cycle to select appropriate clip size and position [14, 23]. Rapid leaflet excursion increases the risk of misinterpreting clip orientation or insertion depth when temporal resolution is insufficient [3].

Consequently, the M-TEER workflow is tightly coupled with continuous real-time echocardiographic guidance and constant bidirectional communication between the echocardiographer and the interventional operator [15, 16]. The 2D and 3D TEE form the backbone of guidance, complemented by MPR for reproducible orthogonal assessment of leaflet capture and device alignment. The CLASP IID trial demonstrated that 3D echocardiographic methodology is the cornerstone of device selection and residual MR prediction in PASCAL (Edwards Lifesciences)-based M-TEER [44].

### Case example

**Fig. 1:**
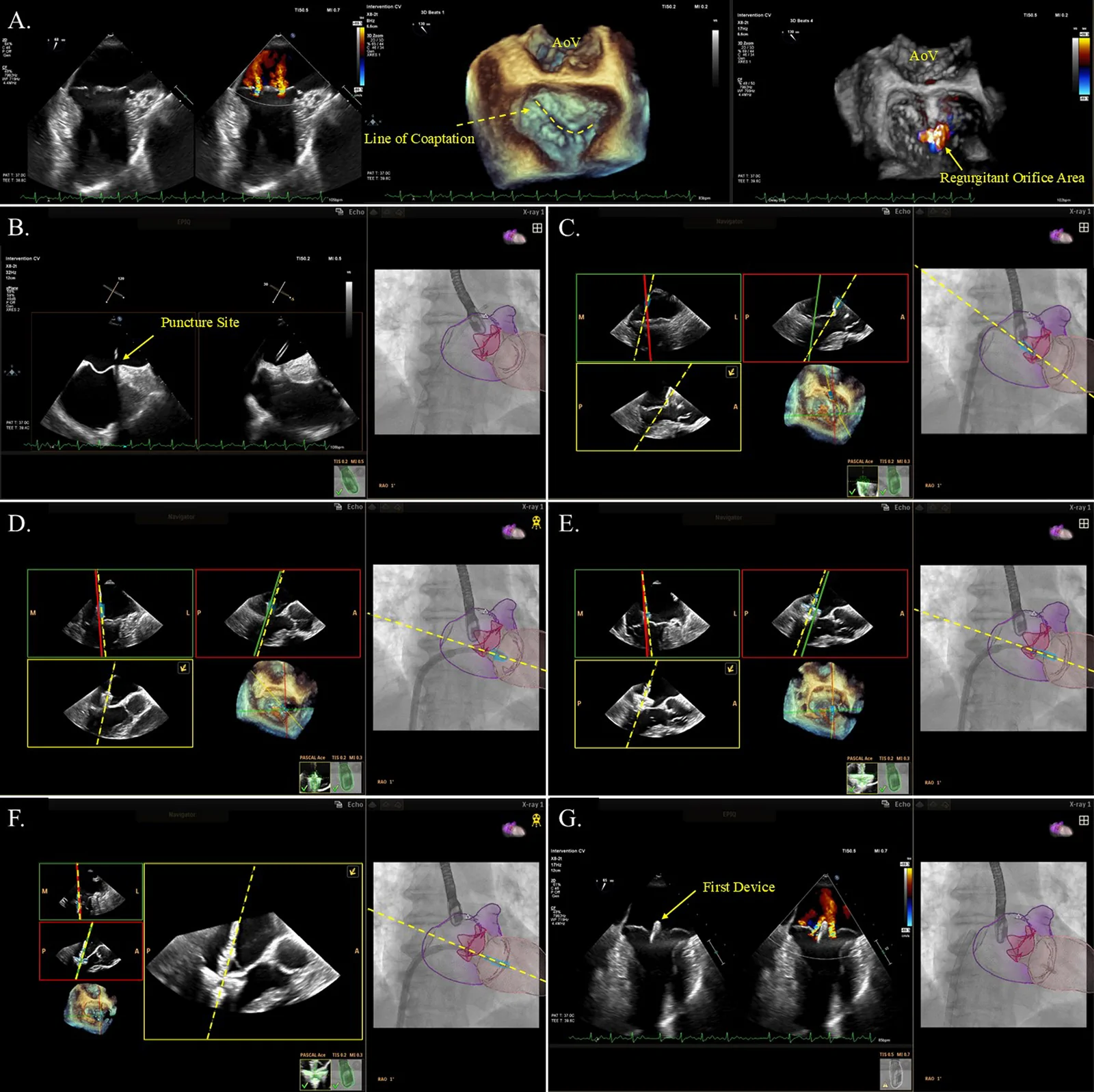
Stepwise echocardiographic guidance during mitral transcatheter edge-to-edge repair (M-TEER). **A** Baseline 2D transesophageal echocardiography (TEE) long-axis view and 3D TEE *en face* view demonstrating asymmetric P2–P3 prolapse with a broad regurgitant orifice. **B** The 2D TEE bicaval view demonstrating appropriate transseptal puncture height above the mitral coaptation plane. **C**–**E** DeviceGuide (EchoNavigator SmartVue, Philips Healthcare) displaying real-time multiplanar reconstruction (MPR) views with continuous visual indicators of device position, trajectory, and rotational orientation relative to the target zone defined at the coaptation line. **F** DeviceGuide with MPR and 3D TEE *en face* view confirming symmetric bilateral leaflet insertion and appropriate device depth prior to TEER device closure. **G** Postdeployment 2D TEE with color Doppler demonstrating a substantial but insufficient mitral regurgitation reduction to grade 2 +. AoV, aortic valve

**Fig. 2:**
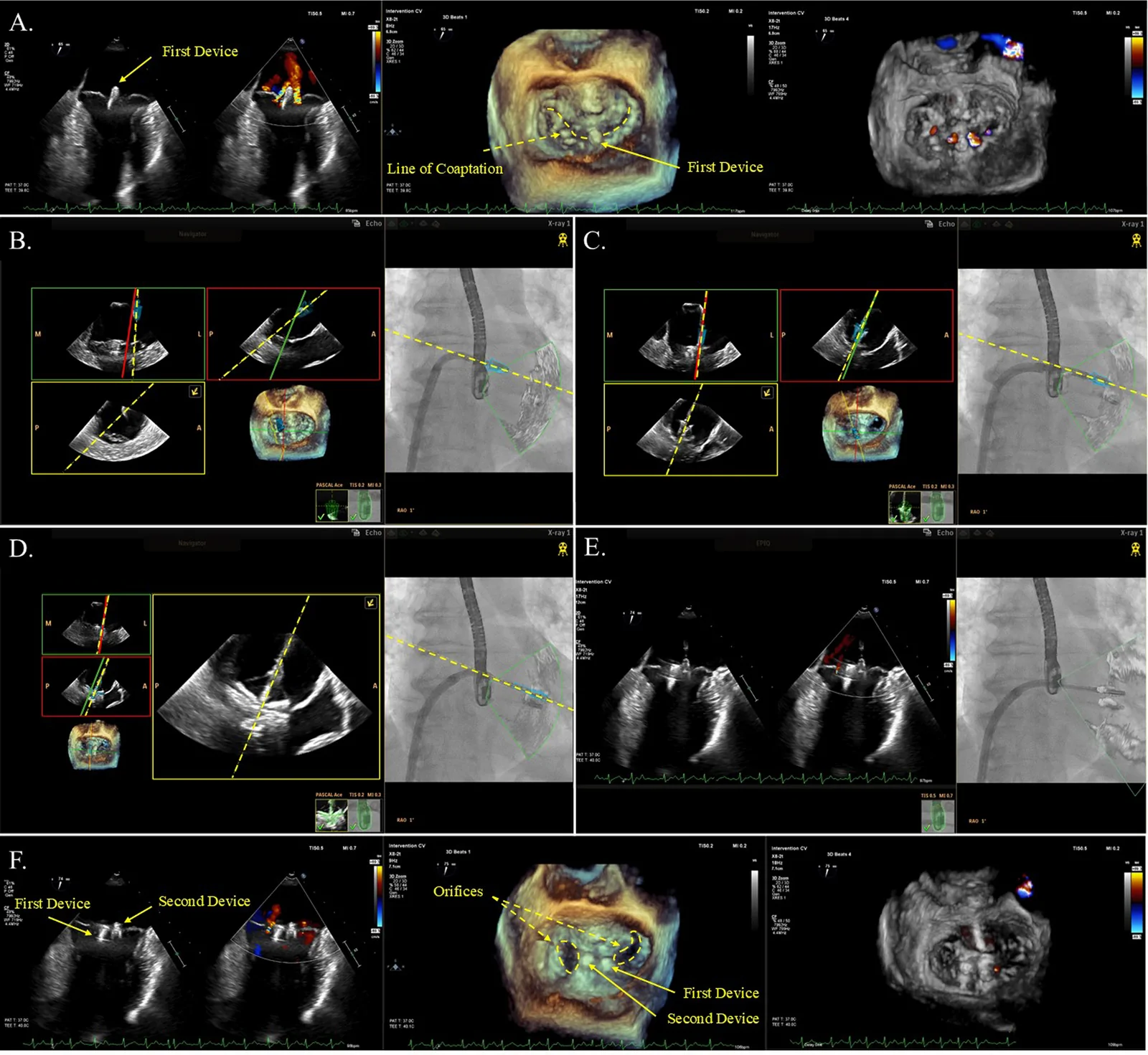
Stepwise echocardiographic guidance during second clip implantation. **A** The 2D and 3D transesophageal echocardiography (TEE) with color Doppler confirming residual mitral regurgitation (MR) following implantation of the first device, prompting a second transcatheter edge-to-edge repair (TEER) device. **B**, **C** Multiplanar reconstruction and DeviceGuide (EchoNavigator SmartVue, Philips Healthcare) views identify the origin of the residual regurgitant jet and defining the optimal position and alignment for the second TEER device relative to the first. **D** DeviceGuide maintaining continuous real-time visualization of the second TEER device trajectory and alignment throughout closure. **E** Echocardiography-fluoroscopy fusion imaging supporting precise spatial orientation and positioning of the second TEER device relative to the first implanted clip within the constrained left atrial workspace. **F** Final postdeployment assessment with 2D and 3D TEE confirming near-complete MR elimination with preserved leaflet mobility

An 82-year-old man with symptomatic severe fibro-elastic degenerative MR involving the full coaptation line underwent M-TEER. Baseline 2D and 3D TEE demonstrated severe MR with asymmetric P2-P3 prolapse and a broad regurgitant orifice (Fig. 1A). Transseptal puncture was guided by 2D TEE to optimize working height above the coaptation plane and confirm posterior fossa ovalis positioning (Fig. 1B). MPR was used to align the delivery system with the commissural axis and confirm approach trajectory. The DeviceGuide platform (EchoNavigator SmartVue, Philips Healthcare) provided target-based MPR views with continuous visual indicators of device position, trajectory, and rotational orientation (Fig. 1C, D). Under trajectory-aligned guidance, the device was advanced across the mitral valve, leaflet insertion was confirmed in orthogonal MPR planes and in 3D *en face* view prior to deployment (Fig. 1E, F). Postdeployment color Doppler demonstrated a substantial but insufficient MR reduction to grade 2 + (Fig. 1G), prompting implantation of a second device (Fig. 2A). MPR and DeviceGuide were used to identify the residual jet origin, and echocardiography-fluoroscopy fusion supported precise spatial orientation of the second device relative to the first clip (Fig. 2B-E). Final assessment confirmed near-complete MR elimination to grade 1 with preserved leaflet mobility and a mean transmitral gradient of 3 mmHg (Fig. 2F). This case illustrates that in full-coaptation-line degenerative disease, a single-device strategy carries meaningful risk of residual MR that structured iterative MPR reassessment alone cannot always anticipate from initial clip positioning. The role of AI-assisted spatial referencing provided important incremental support at the second-device stage: by maintaining continuous orientation within a left atrial workspace already occupied by the first clip, it reduced the cognitive demand that would otherwise have been required through conventional MPR guidance alone. This advantage of AI-assisted orientation in multidevice scenarios will be discussed further.

### Transcatheter mitral valve replacement

#### Annular-level, spatially driven guidance

TMVR focuses on annular-level anatomy and device-tissue interactions involving large, stiff prosthetic systems whose deployment fundamentally alters LVOT geometry [9, 17, 45]. Intraprocedural success depends on accurate spatial navigation, coaxial alignment with the mitral annular plane, controlled implantation depth, full prosthesis expansion, and preservation of LVOT patency. While preprocedural CT remains the reference standard for annular sizing and LVOT risk stratification per the 2025 ESC/EACTS guidelines [4], TEE serves as the irreplaceable tool for real-time intraprocedural guidance, providing dynamic tissue visualization that static CT planning cannot [9, 43, 46, 47].

TMVR presents imaging challenges distinct from M-TEER. The mitral annulus is frequently irregular, elliptical, and partially or circumferentially calcified, rendering fluoroscopic identification of the true annular plane unreliable [45, 46]. Large delivery systems require greater left atrial workspace and offer substantially reduced steerability compared with M-TEER catheter systems, increasing the demand for precise trajectory control from the TSP step onward [9, 14]. Displacement of the anterior mitral leaflet during prosthesis deployment and its interaction with septal geometry critically determines neo-LVOT dimensions and LVOT obstruction risk [43, 46, 47].

High-fidelity multimodal imaging, integrating echocardiography and fluoroscopy, and informed by CT-derived anatomical models, is required to address these complexities [9, 46]. The 3D TEE and MPR provide spatial orientation and depth control during prosthesis advancement and deployment, while echocardiography-fluoroscopy fusion imaging facilitates maintenance of coaxiality throughout the deployment sequence [14, 32].

### Case example

**Fig. 3:**
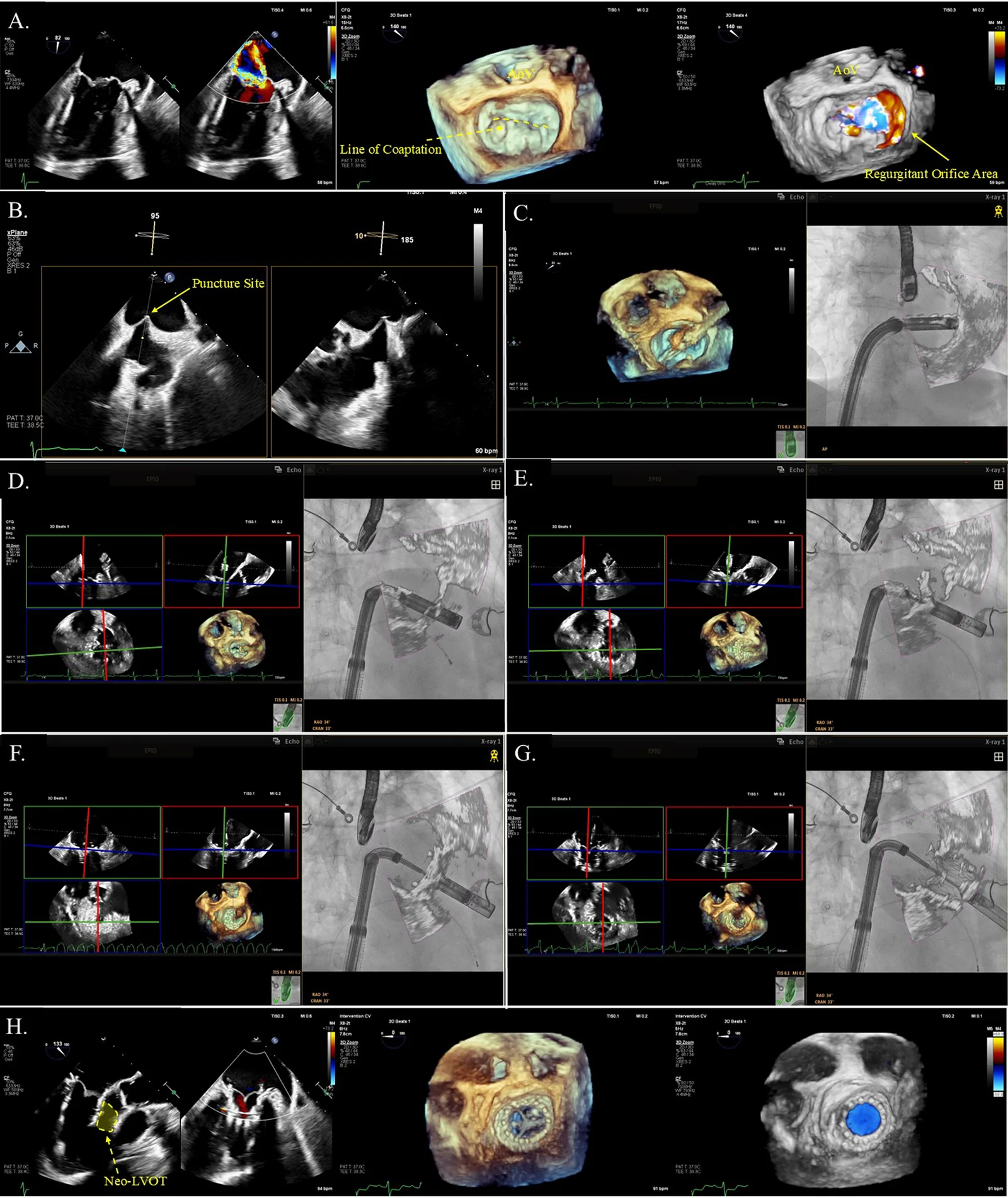
Stepwise multimodal imaging guidance during transcatheter mitral valve replacement (TMVR). **A** The 2D and 3D transesophageal echocardiography (TEE) demonstrate severe mitral regurgitation (MR) with complex posterior leaflet pathology and significant mitral annular calcification. **B** The 2D TEE guidance confirming a posterosuperior puncture site with adequate height for large-delivery-system navigation. **C** The 3D TEE combined with echocardiography-fluoroscopy fusion confirming septal crossing and spatial orientation of the delivery system relative to the annular plane. **D** Real-time multiplanar reconstruction (MPR) providing orthogonal geometric control during intra-atrial navigation; fusion imaging maintaining a shared spatial reference with fluoroscopy. **E**, **F** MPR verification of implantation depth and rotational stability during partial deployment; fusion imaging preserving coaxial orientation. **G** Integrated MPR and fusion imaging confirming stable annular engagement and appropriate prosthesis seating. **H** Postdeployment 3D TEE with color Doppler demonstrating complete MR elimination, absence of significant paravalvular leak, and preserved left ventricular outflow tract flow following intrepid TMVR

An 83-year-old woman with symptomatic severe MR due to posterior leaflet prolapse and significant MAC underwent TMVR with the Intrepid system (Medtronic). Baseline 2D and 3D TEE confirmed severe MR with complex leaflet pathology and a broad regurgitant orifice, supporting selection of annular-level replacement over leaflet repair (Fig. 3A). Transseptal puncture was guided by 2D TEE to achieve a posterior, superior puncture site for large-delivery-system navigation (Fig. 3B). Completion of septal crossing was confirmed using 3D TEE combined with echocardiography-fluoroscopy fusion imaging (Fig. 3C). Intra-atrial navigation was guided by MPR for orthogonal geometric control, with fusion imaging maintaining a shared spatial reference with fluoroscopy (Fig. 3D). During partial deployment, MPR enabled verification of implantation depth and rotational stability, while fusion imaging preserved coaxial orientation relative to the annular plane (Fig. 3E, F). Final prosthesis positioning was confirmed using integrated MPR and fusion imaging (Fig. 3G). Postdeployment 2D and 3D TEE with color Doppler demonstrated complete MR elimination, no significant paravalvular leak, unobstructed LVOT flow, and preserved hemodynamics (Fig. 3H). This case demonstrates that in MAC-associated TMVR, annular irregularity may limit the reliability of fluoroscopic landmarks alone. MPR-centered spatial control therefore becomes the primary echocardiographic reference for annular-level decisions, while fluoroscopy remains essential for device visualization, delivery-system control, and procedural safety. This distinction from the M-TEER paradigm should explicitly inform the design of TMVR imaging workflows and training curricula.

## AI-assisted intraprocedural guidance: roles, evidence, and limitations

AI-assisted guidance represents a conceptual evolution from modality-based imaging toward integrated decision-support in TMVIs [39, 40, 43]. The tools described in this section vary in clinical maturity: the DeviceGuide platform is the only currently cleared implementation for intraprocedural TMVI guidance; the remaining applications discussed are in clinical evaluation or remain investigational, and readers should verify regulatory status for their jurisdiction at the time of use. Rather than introducing novel anatomical information, contemporary AI-assisted tools function as a crosscutting computational layer that organizes, stabilizes, and contextualizes echocardiographic and multimodal imaging data throughout the procedure [40, 43, 48]. Their principal contribution lies in preserving spatial consistency, reducing cognitive burden, and supporting reproducible intraprocedural decision-making across procedural steps and device platforms, while maintaining full operator control and clinical responsibility.

### Spatial consistency and cognitive augmentation

A central contribution of AI-assisted guidance is the preservation of stable geometric reference frames within the highly dynamic intraprocedural environment of TMVIs. Continuous tracking of anatomical landmarks, device position, and predefined spatial targets enables persistent orientation relative to the mitral annulus, leaflet scallops, and adjacent structures across modality transitions and procedural phases [41, 42]. This mitigates cumulative spatial drift, a risk that is particularly consequential in the complex 3D geometry of the mitral apparatus during prolonged procedures or multi-device interventions and reinforces spatial intent without supplanting expert interpretation.

TMVIs simultaneously impose substantial cognitive demands: the echocardiographer must maintain spatial orientation across modalities, track device position in three dimensions, interpret hemodynamic changes in real time, and communicate device adjustments to the interventional operator, often within seconds during active deployment [39, 40]. AI-assisted guidance mitigates this burden by automating repetitive spatial monitoring and reference-frame maintenance tasks, allowing operators to focus on therapeutic execution and higher-order clinical decision-making [49, 50]. Human–machine complementarity theory, well established in surgical and interventional settings, supports this redistribution: effective AI augmentation enhances expert performance by applying algorithmic support to repetitive spatial tracking tasks while enabling expert judgment to focus where it has the greatest clinical impact [40, 49]. This redistribution of cognitive load does not diminish operator expertise, rather, it allows that expertise to be deployed more effectively.

### Procedural reproducibility, standardization, and scalability

The following contributions to reproducibility and training represent conceptually compelling but as yet clinically unvalidated hypotheses. Prospective multicenter data are required before these effects can be regarded as established outcomes. With that caveat stated explicitly, standardized spatial reference frames and alignment criteria provided by AI-assisted guidance may reduce interoperator variability in procedural execution, a relevant consideration as TMVIs expand to more heterogeneous patient populations and as novel devices introduce additional technical complexity [48, 51]. From a training perspective, AI-assisted spatial feedback may shorten learning curves by reinforcing correct spatial relationships during procedural execution, consistent with evidence from other complex interventional specialties [40, 49, 50].

### Procedure-specific prioritization

Although the underlying computational approach of AI-assisted guidance is largely procedure-agnostic, its clinical relevance is shaped by mechanism-specific priorities [39, 40, 48]. In M-TEER, AI guidance may emphasize maintenance of alignment relative to the coaptation line, symmetry during multi-device engagement, and prevention of mediolateral or rotational drift. In TMVR, priorities shift toward accurate definition of the mitral annular plane, maintenance of coaxial alignment of large delivery systems, and continuous spatial awareness of prosthesis-annular-LVOT relationships [9]. This adaptability supports AI as a flexible guidance layer across diverse device platforms rather than a device-specific solution.

### Platform-based clinical implementation

The DeviceGuide platform represents the most clinically advanced current implementation of AI-assisted TMVI guidance. The platform received clearance in 2026 for M-TEER guidance with the PASCAL Ace device (Edwards Lifesciences) and integrates real-time 3D TEE, fluoroscopy, stable MPR reference frames, and continuous AI-assisted device tracking within a unified interface [41, 42]. The regulatory status of specific implementations should be confirmed at the time of clinical use, as approvals in this space continue to evolve.

Biaggi et al. [41] reported a structured single-center experience demonstrating the feasibility of DeviceGuide integration into M-TEER workflows, with observed improvements in spatial consistency and team communication. Hahn et al. [42] described the initial clinical application in a detailed case report. These publications represent encouraging early evidence, but multicenter randomized or prospective comparative data are not yet available.

### Limitations and safeguards

AI-assisted guidance is fundamentally dependent on the quality, stability, and geometric consistency of the underlying imaging data [39, 51]. Errors in acquisition, suboptimal volumetric datasets, or loss of spatial alignment may propagate through AI-derived outputs and visualizations, underscoring the continued requirement for expert imaging oversight and high-quality primary acquisition. Notably, in patients with atrial fibrillation, beat-to-beat variability and volume-compositing limitations reduce the geometric consistency of the underlying 3D datasets on which AI landmark-tracking algorithms depend, potentially compromising the stability of AI-generated spatial reference frames in this specific subpopulation. In practical terms, AI-derived overlays should therefore be regarded as conditional decision-support tools. They should be actively verified against conventional 2D and 3D TEE, Doppler, and fluoroscopy, and temporarily disregarded until recalibration or reacquisition when image quality deteriorates, acoustic shadowing or device artifact obscures the target anatomy, co-registration drift is suspected, or the AI output no longer matches the observed leaflet, device, or flow pattern. AI does not replace clinical judgment, nor does it eliminate the need for echocardiographic expertise [49].

Appropriate safeguards include transparency of AI outputs, operator ability to override AI-generated recommendations, and maintained awareness of system limitations during time-critical procedural decisions [40, 49]. Prospective multicenter studies evaluating the impact of AI-assisted guidance on procedural efficiency, safety, and clinical outcomes across diverse anatomies, operator experience levels, and device platforms remain essential to define the independent clinical value of these tools, and to distinguish genuine outcome benefit from workflow convenience.

## Future directions: toward platform-based multimodal imaging guidance

Intraprocedural imaging guidance for TMVIs is undergoing a fundamental transition from procedure- and device-specific approaches toward integrated, platform-based guidance environments. This evolution is driven by increasing procedural complexity, expanding device diversification, and the need for reproducible, scalable imaging strategies across heterogeneous patient populations and operator experience levels [2, 9]. The technological directions outlined below span different time horizons: deeper platform integration and AI implementation are already partially realized in current clinical environments, augmented reality and finite element modeling remain in early evaluation, and fully automated guidance represents a longer-term aspiration.

In this emerging paradigm, echocardiography is expected to remain the central imaging modality, providing continuous real-time visualization of anatomy, device-tissue interaction, and hemodynamic response. Its role will be further enhanced through integration with fluoroscopy, CT-derived spatial models, and AI-assisted approaches that maintain geometric consistency across procedural steps and modality transitions [9, 15, 41]. By reducing fragmentation between imaging modalities and preserving continuity of spatial reference from access and navigation through deployment and postprocedural assessment, integrated platforms have the potential to improve procedural efficiency, enhance team communication, and reduce cognitive burden during complex interventions.

Among near-term technological developments, augmented reality overlay systems projecting echocardiographic or CT-derived anatomical models into the operator’s visual field are in early clinical evaluation and may further reduce the cognitive burden of spatial translation between modalities [52]. However, integration of CT-derived anatomical models into the live procedural environment remains technically demanding. Static CT datasets must be co-registered with real-time TEE and fluoroscopy, despite differences in rhythm, loading conditions, respiratory phase, patient positioning, and procedural manipulation. Current workflows therefore still require expert manual verification and recalibration rather than fully automated alignment. Looking further ahead, real-time finite element modeling of prosthesis-tissue interactions, informed by live imaging data, could enable predictive assessment of deployment outcomes before device release, a concept with established methodological foundations in computational cardiac mechanics [53]. Fully automated echocardiographic plane identification may ultimately support procedural standardization in lower-volume centers, though this requires validation across the anatomical heterogeneity characteristic of TMVI populations.

The expanding application of TMVI principles to tricuspid valve interventions represents an important adjacent development. As transcatheter tricuspid edge-to-edge repair and replacement enter clinical practice, supported by evidence from the TRILUMINATE Pivotal trial, CLASP TR early feasibility study, and TRISCEND II trial [54–56], the imaging frameworks developed for mitral interventions will require adaptation to the distinct anatomical challenges of the tricuspid apparatus, including its more anterior position, greater anatomical variability, and stronger dependence on right ventricular geometry. Whether the proposed mitral paradigm (leaflet-level temporally driven vs. annular-level spatially driven) translates directly to tricuspid interventions remains an open question. Tricuspid transcatheter edge-to-edge-repair shares the leaflet-engagement logic of M-TEER, but the three-leaflet anatomy, more complex subvalvular apparatus, and anterior cardiac position may demand a distinct spatial and conceptual framework rather than a simple transposition of mitral imaging principle.

The adoption of platform-based imaging will require evolution in training pathways for both interventional operators and structural echocardiographers, shifting emphasis toward proficiency in integrated imaging environments rather than modality-specific expertise alone [49, 51]. While AI and platform tools may facilitate learning curve reduction by stabilizing spatial orientation and standardizing procedural steps, their effective use will remain dependent on a strong foundation in echocardiographic acquisition, interpretation, and intraprocedural decision-making.

Several practical challenges must be acknowledged. Implementation of advanced imaging platforms may be constrained by resource availability, procedural costs, and vendor dependency, as well as by institutional expertise disparities between high-volume structural heart centers and emerging programs [51]. Prospective multicenter studies demonstrating the impact of integrated imaging and AI-assisted guidance on procedural outcomes, complication rates, and long-term clinical benefit across diverse clinical settings will be prerequisites for broader adoption. Standardization of imaging protocols and validation will be equally essential.

Collectively, these developments point toward a paradigm in which intraprocedural imaging evolves from a collection of complementary tools into a cohesive, echocardiography-centered platform integrating multimodal imaging and decision-support technologies within a unified procedural environment. In this landscape, the role of intraprocedural imaging extends beyond visualization, acting as a central determinant of procedural strategy, precision, safety, and scalability across the full spectrum of transcatheter mitral valve therapies.

## Conclusion

Intraprocedural echocardiographic guidance has evolved from a supportive visualization modality into a central determinant of procedural success in TMVIs. The increasing anatomical complexity, expanding device landscape, and advancing procedural strategies necessitate coordinated imaging approaches that provide continuous spatial orientation, dynamic assessment of device-tissue interactions, and real-time hemodynamic evaluation across all procedural phases.

The explicit distinction between leaflet-level, temporally driven guidance for M-TEER and annular-level, spatially driven guidance for TMVR is not merely taxonomic, it carries direct and practical consequences for imaging workflow design, echocardiographer-operator communication, and procedural training curricula. Effective intraprocedural imaging is objective-driven rather than modality-centric, and the failure to align imaging strategies with procedural mechanisms is a source of avoidable risk.

Within this framework, the modalities described in this review function not as interchangeable alternatives but as complementary layers of coherent, objective-driven guidance architecture, one whose effectiveness depends on their structured integration rather than their individual application. The 2D and 3D TEE provide complementary real-time guidance, MPR serves as the geometric backbone bridging temporal and spatial imaging precision, echocardiography-fluoroscopy fusion imaging supports crossmodal spatial integration, 3D ICE expands procedural accessibility in selected patients, and AI-assisted tools add a computational guidance layer enhancing spatial consistency without replacing expert judgment.

The transition from modality-based toward integrated, platform-based guidance environments represents the defining direction of next-generation TMVI echocardiography. Realizing this potential will require technological development, rigorous prospective validation, protocol standardization, and a renewed emphasis on training that reflects the multimodal, integrated nature of modern structural echocardiographic practice.

## Data Availability

No datasets were generated or analysed during the current study.

## Abbreviations

AI: Artificial intelligence
CT: Computed tomography
ESC: European Society of Cardiology
EACTS: European Association for Cardio-Thoracic Surgery
ICE: Intracardiac echocardiography
JACC: *Journal of the American College of Cardiology*
LVOT: Left ventricular outflow tract
MAC: Mitral annular calcification
MPR: Multiplanar reconstruction
MR: Mitral regurgitation
M-TEER: Mitral transcatheter edge-to-edge repair
SCAI: Society for Cardiovascular Angiography & Interventions
TEE: Transesophageal echocardiography
TMVI: Transcatheter mitral valve intervention
TMVR: Transcatheter mitral valve replacement
TSP: Transseptal puncture
